# Possible Clinical Evidence for a Glossopharyngeal Afferent Pathway in Human Hiccup Reflexes: A Report of Two Cases

**DOI:** 10.7759/cureus.106190

**Published:** 2026-03-31

**Authors:** Yuki Kojima, Tsukasa Kondo

**Affiliations:** 1 Anesthesiology, Asahi General Hospital, Asahi, JPN; 2 Emergency and Critical Care Medicine, Yuai Memorial Hospital, Koga, JPN

**Keywords:** dental procedure, glossopharyngeal nerve, glossopharyngeal nerve block, hiccup, reflex

## Abstract

Although hiccups are a reflex involving diaphragmatic contraction and glottic closure, the afferent pathway involved in humans remains unclear. We report the cases of two adult patients who developed hiccups during intravenous sedation for dental procedures. In both cases, midazolam administration immediately provoked persistent hiccups. Ultrasound-guided selective glossopharyngeal nerve block with local anesthetic resulted in immediate and complete hiccup cessation, allowing the uneventful continuation of dental procedures. Both patients had previously received midazolam without experiencing hiccups, suggesting that the drug alone was insufficient to trigger the response. Hence, reflex activation by pharyngeal stimulation from saliva or secretions may be necessary. These observations highlight a potentially under-recognized afferent pathway with diagnostic and therapeutic implications. The glossopharyngeal nerve contributes to hiccup generation in humans. Therefore, understanding hiccup pathophysiology may enable the management of underlying conditions in addition to symptom relief.

## Introduction

Hiccups are a pathological respiratory reflex characterized by spasm of one or both sides of the diaphragm, resulting in sudden inspiration and closure of the glottis. The accessory respiratory muscles may also be involved [1]. Hiccups are widely recognized as an important clinical sign among healthcare professionals, as they can present as a symptom of various conditions; however, many aspects of their underlying causes and detailed mechanisms remain unclear. This phenomenon represents a paradoxical movement in which the diaphragm promotes inspiration, whereas the glottis simultaneously prevents air entry. Because hiccups are observed even in neonates and fetuses, they are considered an innate motor pattern [2]. Thus, a hiccup can be physiologically defined as a reflex, rather than a mere diaphragmatic spasm.

Bailey first proposed the concept of a "reflex arc" responsible for hiccup generation in 1943 [3]. The afferent impulses in this arc were hypothesized to be transmitted via the vagus nerve, phrenic nerves, or sympathetic fibers. The implicated central nervous system components include the upper spinal cord, medulla oblongata near the respiratory centers, reticular formation, and hypothalamus [3,4]. Despite the presentation of numerous hypotheses, a lack of methods to verify them has meant that hiccups are one of the reflexes that have long remained poorly understood.

By conducting experiments in cats, Arita et al. demonstrated that the hiccup center is located within the reticular formation near the nucleus ambiguous [5]. The efferent pathway clearly involves the phrenic nerve, which induces diaphragmatic contraction, and the recurrent laryngeal branch of the vagus nerve, which induces glottic closure. However, Bailey’s original hypothesis, that the vagus and phrenic nerves constitute the afferent pathway [3], has not been experimentally validated.

In 2003, Kondo et al. reported that hiccups could be induced in cats by stimulating the pharyngeal branch of the glossopharyngeal nerve [6]. They further demonstrated that hiccups were evoked only when stimulation was applied during inspiration, that train pulses were required as single-pulse stimulation was insufficient, and that the glossopharyngeal nerve did not directly activate the diaphragm or vocal cords but instead operated through a reflex center. Collectively, these findings indicate that the pharyngeal branch of the glossopharyngeal nerve constitutes the sole afferent pathway mediating hiccups. Nevertheless, this concept remains largely unacknowledged, likely because (i) clinicians find it counterintuitive that pharyngeal stimulation can induce hiccups and (ii) definitive evidence confirming the existence of an equivalent reflex pathway in humans is lacking.

Herein, we describe two clinical cases that suggest that the pharyngeal branch of the glossopharyngeal nerve is indeed involved in hiccup generation in humans. Additionally, we propose a hypothesis to clarify the afferent pathway of hiccups.

## Case presentation

Case 1

A 40-year-old woman (height 153 cm, weight 69 kg) with intellectual disability and a severely abnormal gag reflex was referred to our hospital for dental caries treatment under intravenous sedation. She was not taking any medications and was able to communicate clearly. For such patients, safe and effective anesthetic management can be achieved by administering sedation with midazolam or propofol in conjunction with an ultrasound-guided selective glossopharyngeal nerve block (UGSGNB). In the present case, intravenous midazolam (5 mg) was administered first. 

**Figure 1:**
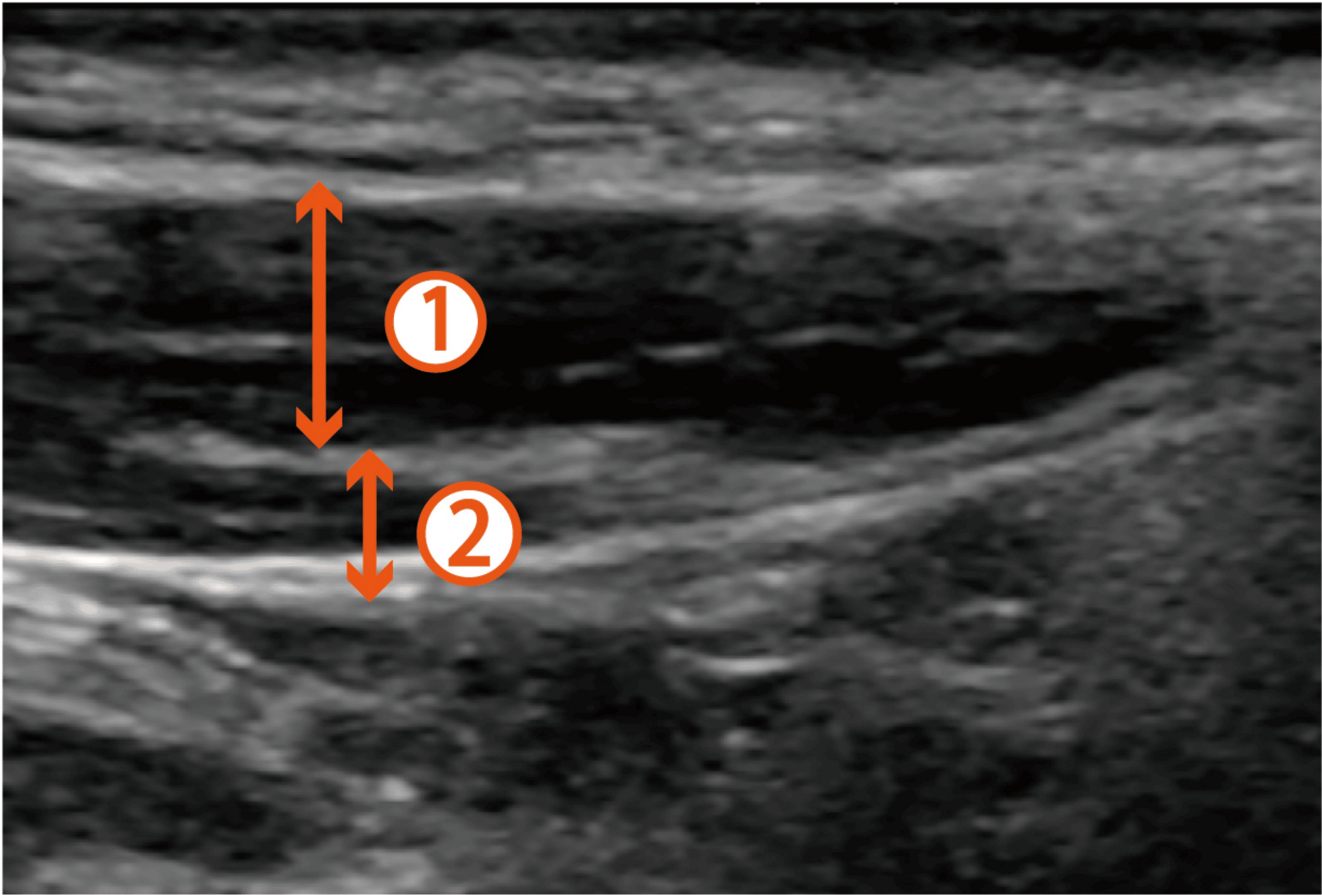
Ultrasound-guided selective glossopharyngeal nerve block in Case 1. After identification of the sternocleidomastoid muscle, a linear ultrasound probe was set parallel to the sternocleidomastoid muscle. 1: Sternocleidomastoid muscle; 2: Stylohyoid muscle.

The patient developed hiccups shortly after sedation onset. Because hiccups are a known adverse effect of midazolam, the episode was diagnosed as drug-induced hiccups. Hiccups began approximately two minutes following midazolam administration. The level of sedation was −2 on the Richmond Agitation-Sedation Scale (RASS) [7], and there was no stimulation to the airway or pharynx. To control the abnormal gag reflex, UGSGNB was performed with 2 mL of 0.2% ropivacaine (Figure 1). This immediately abolished the hiccups, and the dental treatment was completed uneventfully (Video). The hiccups ceased within approximately 30 seconds of performing UGSGNB. The hiccups did not recur during surgery. No abnormalities were noted after recovery from anesthesia. This patient underwent several dental treatments under intravenous sedation and experienced three hiccups in total. At each instance, the symptoms resolved with UGSGNB.

**Video 1:** Hiccup management in Case 1. Hiccups were induced following midazolam administration. While the patient was experiencing hiccups, an ultrasound-guided selective glossopharyngeal nerve block was performed bilaterally. The video recording demonstrates the immediate disappearance of hiccups after the block is performed. For this procedure, the patient turned their face toward the clinician, and a linear ultrasound probe was placed in the neck to check the surrounding target structures. Following identification of the sternocleidomastoid muscle, a probe was placed caudal to the mandibular ramus. The stylohyoid muscle was visualized by tilting the linear ultrasound probe toward the mandibular ramus. A needle was then inserted deeply under the stylohyoid muscle through the sternocleidomastoid muscle using an out-of-plane approach.

Case 2

A 23-year-old woman (height 155 cm, weight 50 kg) with dental phobia was referred to our hospital for wisdom tooth extraction under intravenous sedation. She had no history of serious illness, was currently healthy, and was not taking any medication. She developed hiccups following intravenous sedation with midazolam (5 mg). Hiccups began approximately three minutes after midazolam administration. The level of sedation was −2 on the RASS, and there was no stimulation to the airway or pharynx.

**Figure 2:**
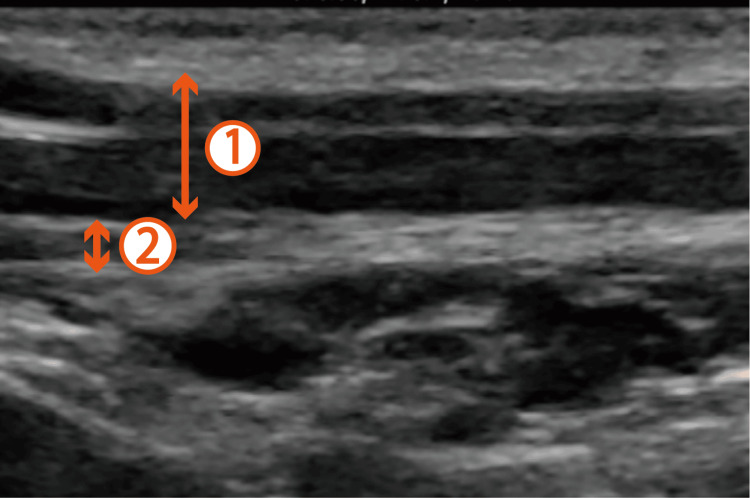
Ultrasound-guided selective glossopharyngeal nerve block in Case 2. The sternocleidomastoid muscle and the stylohyoid muscle can be observed behind the mandibular ramus. 1: Sternocleidomastoid muscle; 2: Stylohyoid muscle.

UGSGNB with 1% lidocaine was performed, resulting in instantaneous hiccup cessation, enabling the uneventful continuation of the procedure (Figure 2). The hiccups ceased within approximately 30 seconds of performing UGSGNB. The hiccups did not recur during surgery, and no abnormalities were noted after recovery from anesthesia. This patient underwent several dental treatments under intravenous sedation and experienced two hiccups in total. Both times, the symptoms resolved with UGSGNB.

Neither patient had previously developed hiccups during sedation with midazolam, indicating that midazolam alone was insufficient to induce them and that an additional trigger, likely pharyngeal stimulation by saliva or secretions, was required.

## Discussion

Alternative explanations for the cessation of hiccups should be considered in these two cases, including spontaneous resolution, changes in sedation depth, non-specific reduction of pharyngeal stimulation, or migration of the local anesthetic to surrounding structures. Further, the absence of statistical analysis in these two cases must be acknowledged. Nevertheless, we consider that the most plausible contributing factor is the effect of UGSGNB.

UGSGNB blocks the pharyngeal and tonsillar branches of the glossopharyngeal nerve [8]. Apart from suppressing abnormal gag reflexes, this nerve block is applied for postoperative analgesia following tonsillectomy and root of tongue surgery [9]. Hiccup cessation after UGSGNB in the two cases presented in this report strongly suggested the involvement of the pharyngeal branch of the glossopharyngeal nerve in the hiccup reflex, providing evidence that the reflex pathway discovered by Kondo et al. in cats [6] also exists in humans.

**Figure 3:**
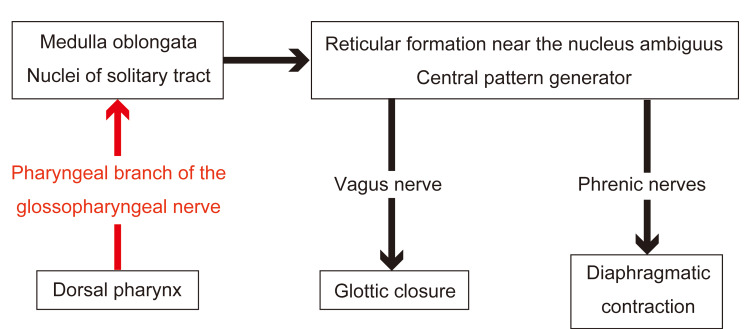
Proposed reflex arc of hiccups in humans. The efferent pathway involves the phrenic nerve and the recurrent laryngeal branch of the vagus nerve. The pharyngeal branch of the glossopharyngeal nerve was proposed as the afferent pathway. Figure Credit: Authors; created using Adobe Illustrator (Adobe Inc., San Jose, California, United States)

Notably, both patients had previously undergone sedation with midazolam without developing hiccups. This suggested that drug administration alone was insufficient to induce hiccups; although this remains only a hypothesis, pharyngeal stimulation by saliva or other secretions may have served as the trigger, supporting the hypothesis that hiccups are mediated by glossopharyngeal afferents (Figure 3).

A literature review revealed substantial evidence suggesting the involvement of the glossopharyngeal nerve in hiccup pathogenesis. Hiccups occur more frequently in association with infections, gastroesophageal reflux disease, and pneumonia [10-12]. They may also arise during bronchoscopy or dental procedures under sedation [13,14]. Intranasal vinegar can reportedly alleviate intractable hiccups in children [15]. Although specific nerve block techniques have not been described in detail, these therapeutic approaches further support the potential role of the glossopharyngeal nerve in hiccup generation. Methods to suppress hiccups include drinking cold water, gargling, cervical compression, and gently pulling the tongue [16]. As these approaches involve the stimulation of areas innervated by the glossopharyngeal nerve, our findings may provide evidence to support these interventions.

Advancing our understanding of the hiccup mechanism may significantly contribute to elucidating hiccup pathophysiology and inhibiting its triggers. Delineation of the physiological mechanism of hiccups will enable the identification and treatment of the underlying conditions or lifestyle factors that provoke them, in addition to providing symptomatic relief. Hiccups are often associated with systemic disorders, such as metabolic or renal diseases [17]; however, their pathophysiological basis remains poorly understood. Therefore, further investigation into the hiccup reflex mechanism is essential. In cases in which hiccups cannot be explained by reflex pathways, alternative interpretations must be sought. Performing UGSGNB in the presence of hiccups requires a higher level of technical skill than usual. As hiccups are involuntary movements, it is essential to confirm that there are no critical structures surrounding the target area before performing a nerve block.

We believe that our hypothesis provides a meaningful perspective for patients experiencing hiccups as well as clinicians striving to manage them.

## Conclusions

The immediate and reproducible cessation of hiccups following ultrasound-guided selective glossopharyngeal nerve block in two independent cases provides possible clinical evidence that the pharyngeal branch of the glossopharyngeal nerve functions as a key afferent limb of the hiccup reflex in humans. These observations suggest that the glossopharyngeal-mediated reflex pathway is not only theoretical but physiologically operative in clinical settings.

Our findings further indicate that hiccups may require both central susceptibility and peripheral pharyngeal stimulation, offering a plausible explanation for sedation-associated hiccups and their variability among individuals. Recognition of this afferent pathway has important clinical implications, as it may inform novel diagnostic and therapeutic strategies, including targeted nerve block interventions for intractable hiccups. Further physiological and clinical investigations are warranted to confirm this reflex mechanism and clarify the precise neuroanatomical integration of glossopharyngeal afferents within the human hiccup reflex arc. A clearer understanding of this pathway may ultimately contribute to more rational and mechanism-based management of hiccups.
